# A Structured and Flexible Language for Physical Activity Assessment and Characterization

**DOI:** 10.1155/2013/420916

**Published:** 2013-09-25

**Authors:** Pedro Silva, Maria Teresa Andrade, Pedro Carvalho, Jorge Mota

**Affiliations:** ^1^CIAFEL, Faculty of Sports, University of Porto, 4200-450 Porto, Portugal; ^2^INESC Porto, University of Porto, 4200-450 Porto, Portugal

## Abstract

Developing more accurate assessments of physical activity (PA) and sedentary behavior (SB) is an important public health research priority. Assessing PA and SB is challenging in all segments of the population, but it is especially difficult in children due to cognitive limitations and more sporadic and intermittent activity patterns. Moreover, they are influenced by several factors including temporal-spatial constraints and social conditions. To accurately assess PA and SB, it is essential to clearly define methods for describing all these factors. The goal of this paper is to potentiate advances in the field by proposing a base ontology for characterizing physical activity, sedentary behavior, and the context in which it occurs. The ontology would establish a flexible base language to facilitate standardized descriptions of these behaviors for researchers and public health professionals.

## 1. Introduction

Epidemiological studies have demonstrated that regular physical activity (PA) is truly a “medicine” with proven efficacy to reduce morbidity and mortality associated with chronic disease [1]. Being physically active is a major contributor to one's overall physical and mental well-being [2]. The literature indicates that PA is influenced by a large group of factors, including, environmental, social, psychological, and cultural ones [3–6]. However, methods are not available to systematically study the complex interactions that influence PA and sedentary behavior (SB). To advance public health research, it is important to develop more robust methodologies. 

Measuring PA and characterizing the context under which the PA occurs are a challenging task. A number of studies have evaluated PA patterns with objective activity monitors, but few studies have examined contextual variation in PA and sedentary activity [7–9]. Recent research efforts have sought to overcome these limitations, and a number of different instruments have been used: daily logs; diaries; questionnaires; direct observation. 

Different areas are needed to tackle the global pandemic of physical inactivity because multidisciplinary work are essential [10]. Moreover, as Hallal et al. [11] stated, “technological advancements that nudge us towards physical inactivity make it urgently necessary to take actions. More of the same (in terms of research and practice) will not be enough.” The challenge is to turn these technological advancements from only user friendly to “health friendly.” More recently, solutions based on global positioning system, (GPS) geographical information system, (GIS) smartphones, and electronic ecological momentary assessment (EMA) have been introduced as these approaches offer considerable advantages in terms of data processing and communication technologies. PA is one of many health behaviors that have the potential to change substantially as a result of increasing availability of information and communication technologies and of technology-based interventions [12].

While many methods have been tested, direct observation (DO) remains as a criterion (gold standard) measure for evaluating the context of PA and SB. Because behaviors are analyzed at the location by a trained observer, DO avoids some limitations of subjective methods (such as the recall of information) and objective methods (such as the reactivity to the devices) [13]. At this time, DO techniques provide the only approach that can objectively code both the behavior and the context in which it occurred. Despite the advantages, systematic observation methods are underused by researchers [14]. The main reason for this is the shortcomings associated with DO methods. The most significant limitation is the cost and burden associated with collecting and processing the data. This barrier typically leads to a limited scope or amount of observation data, bringing into question whether data from those samples are adequate for that setting or whether they can be generalizable. Another major limitation is that observers must be properly trained to be objective and nonjudgmental. Steps must be taken to ensure that they maintain their skills over time, and there is the possibility of observed people behaving differently when an observer is present (i.e., reactivity). 

These limitations have proven difficult to overcome, but new advances in automated video-based processing techniques offer considerable promise in this area. Advances in sensor, communications, and computer technologies favor their introduction in the PA assessment problem. Comprehensive surveillance systems are crucial to advance public health. The development and introduction of such a comprehensive system pose challenges and are dependent on the capacities and resources available. Yet having such PA information will serve to improve investment of scarce resources, increase accountability, and help to make efficient and effective investments [10]. While the use of devices such as GPS or accelerometers has provided interesting results, their usage presents a burden to participants and has a number of significant limitations for field-based research. The most significant limitation is the inability to extract meaningful contextual information. Advanced video-based tracking systems have been shown to have considerable promise in surveillance and security applications, and these same techniques can be applied to enhance the monitoring of PA and SB. The video has the advantage of being nonintrusive and enabling more complete contextual characterizations. The introduction of new technologies has contributed to a finer temporal resolution of the observations while decreasing the burden in the human observer. Moreover, an increase in automation has improved the objectivity of the process and extended the type/diversity of gathered data. These attributes directly overcome some of the stated limitations of current DO tools. If similar technology is applied to PA and SB assessment, it would be possible to directly (and automatically) code these behaviors. 

Before this can be done, it is necessary to first develop a systematic way to code the various classes and types of behavior. This is essential since the volume and diversity of collected data can only be adequately managed if the data can be properly represented, using well-established rules and formats. The absence of a common language to represent this information and interpret the individual behavior is presently an obstacle in this research field. Reinforced by the absence of standardized instruments suitable for international use by the researchers [15]. Its existence would foster the exchange of information between researchers and facilitate its reuse and analysis. This is particularly significant with the increased granularity of the supported classes. 

Ontologies can be a solution to create a standard and interoperable language for specific application domains. According to Grüninger and Lee [16], the use of ontologies can serve three purposes: (1) to support communication, between machines, between humans, and between humans and machines; (2) for knowledge reuse as, for example, shared repositories of technical documentation; (3) for computational deduction, enabling the extraction of new knowledge from existing data as, for example, in internal structure analysis. The development of a clear ontology for capturing the context and diverse aspects of PA and SB is a priority for advancing the development of more automated DO tools. Continued improvement in monitoring behavior would help to guide development of policies and programs to increase activity levels and to reduce the burden of noncommunicable diseases [15]. To the author's knowledge, there is currently no ontology available to facilitate the systematic study of PA and SB. Therefore, the present paper will propose an ontology for assessing PA and SB (OPA ontology), containing concepts that can capture knowledge about the context in which the behavior is being performed. The goal is to establish a common ground for researchers assessing behavior and its context using different instruments.

## 2. Methods 

### 2.1. PA and SB Categorization

 An individual activity profile is characterized by a complex matrix of behaviors that take place in a range of social contexts, each with its own set of physiological, psychological, and sociological determinants and outcomes. Sallis et al. [17] stated that the wide range of correlates supports the application of ecological models of behavior to improve understanding of the influences on PA. Moreover, variables within individuals, such as psychological and biological factors, are widely studied, as are interpersonal variables. Environmental, policy, and global variables are less studied but are thought to have widespread effects. The combination and interaction of factors and at these levels are expected to influence PA [6]. Hence, PA behavior is best described as a profile rather than a single entity or indicator. 

**Figure 1:**
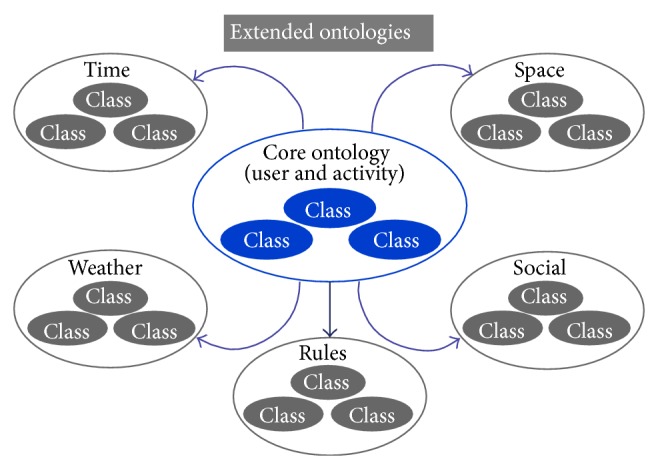
Conceptual profile of the factors influencing physical activity assessment and characterization.

Figure 1 presents a profile that does not pretend to be a socioecological model, but rather a conceptual diagram to simplify the characterization of physical activity and sedentary behavior and its context. In the center, there are individual characteristics that shape self-behavior. The interception of the other domains provides the context where the behavior occurs and how the activity can be characterized or classified.

Another aim of the model (Figure 1) is to facilitate the assessment of PA and SB since it focuses on fundamental domains of these behaviors; that is, they are influenced by space, time, and social factors. It supports examination of problems similar to the way they occur in a natural setting, with simultaneous influences of multiple factors. Since physical activity is multifaceted, it makes good theoretical sense to evaluate those facets using these factors. DO methods are considered to be a gold standard for capturing PA and SB classification, but, as previously stated, they have important shortcomings. The need to observe the activity *in situ* requires a small number of classes of classification to enable the process. As a result, state-of-the-art methods cannot capture all the factors involved. Therefore, monitoring individual level and microenvironment social inequalities is crucial for evaluating the effects of programs and policies and providing an insight into whether current efforts should be continued or modified [18]. The dilemma in examining PA in a theoretical framework is balancing parsimony with comprehensiveness [19]. Research needs improved measures of exposure (correlates), objective PA measures, prospective designs, and advanced data modelling to assess causal determinants rather than just associations between variables [6]. 

### 2.2. OPA: A Common Language for PA and SB Classification

To integrate all the previous concepts, we propose the OPA ontology. A useful conceptual definition of ontology can be consulted here [20]. The aim of an ontology is to capture and to create knowledge models that can be reused and shared between applications or groups of people [21]. The availability of a PA and SB ontology, defining a common vocabulary for researchers in this area, would provide an important tool for information sharing and reuse, thus increasing the opportunity for advances in the domain. 

An ontology can be seen as collection of terms from a vocabulary, capturing into a formal model and enabling automated processing of that model, common understanding and knowledge about a given domain knowledge or real-world situation populated by resources. The fundamental blocks of an ontology are classes, individuals, and properties. A class represents a category or a group of resources sharing common characteristics. Individuals are specific resources that belong to a given category or class; that is, they are instances of classes. Finally, properties enable describing the resources. They can be used to establish relations between classes and individuals or between individuals and literal values associated with them [20]. In the present domain of knowledge—assessment of PA and SB—the resources in the ontology would be persons and groups or categories of persons and activities and categories of activities. Additionally, given the intention of characterizing the activity not only through its type (e.g., sitting, running, jumping, etc.) but also with the information about the context under which that type of activity is taking place, resources would also be concepts, and their instances are associated with the time, space, and social dimensions.

To develop an ontology, it is necessary to choose a language to represent it. One of the most commonly used languages is Web ontology language (OWL) [22, 23]. OWL is a standard specified by the World Wide Web Consortium (W3C) [24] adding new capabilities to the standards previously released by the W3C in pursuit of the Semantic Web paradigm, notably, Extensible Markup Language (XML), Resource Description Framework (RDF), and RDF Schema (RDFS). RDF is an XML-based language used to represent information on the Web. RDFS extends the scope of application of RDF, by enabling the development of specific metadata vocabularies and describing relationships among resources in terms of named properties and values. Comparing with RDFS, OWL extends the use of properties by enabling the use of statements regarding properties and by imposing restrictions on how properties behave. OWL was designed to allow any software or hardware system (or any human) to be able to process the information contained in the ontology, reason about it and unambiguously interpret it, and infer the original high-level concepts. 

**Figure 2:**
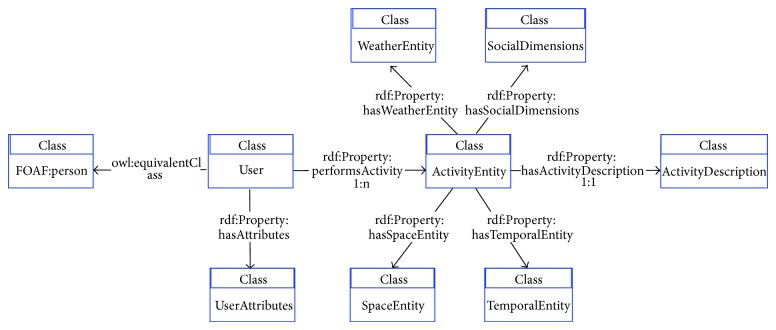
Conceptual diagram of the base classes intended for physical activity and inactivity classification.

Based on these classes and on the model proposed in Figure 1, we define an ontology hierarchy to be used as a common language for researchers involved in PA and SB analysis and classification.  Figure 2 presents the proposed OPA ontological model. The core ontology is composed of the defined generic classes for activity classification and for the individual conducting the activity. Those classes follow very closely the widely accepted compendium for PA by Ainsworth et al. [25]; the compendium is used globally in a variety of situations, for example, to quantify the energy cost of PA and SB in adults for surveillance activities [26]; in research studies and in clinical settings; to write PA recommendations; to assess energy expenditure in individuals. Connected to this base ontology, extended ontologies are used to allow capturing knowledge external to the activity but very likely to have a decisive impact on the way activity is conducted. These extended ontologies incorporate concepts that represent factors related to the dimensions “Time,” “Space,” and “Social.” They can be seen as the representation of the context under which the activity is being performed and will permit standardized comparisons of correlates, with similar measures in high-income and low-income countries, that take into account strengths of different correlates and an investigation of cultural and country-level factors [6].

**Figure 3:**
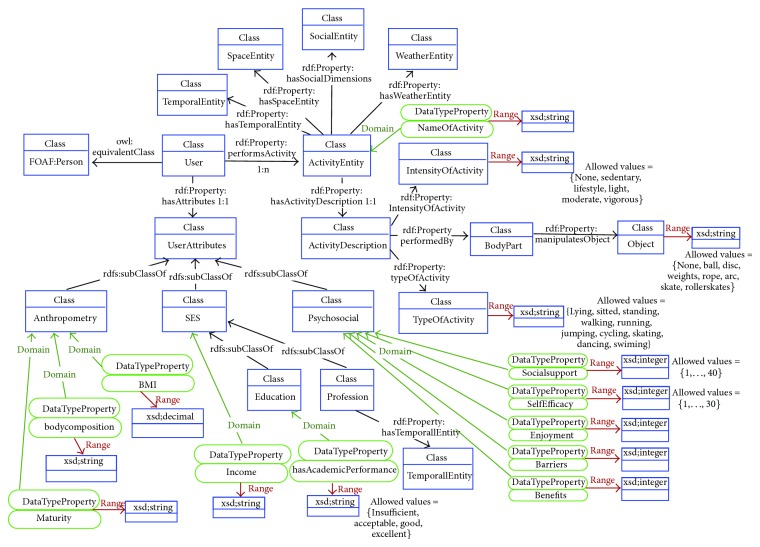
Conceptual diagram to characterize an individual performing physical activity.

**Figure 4:**
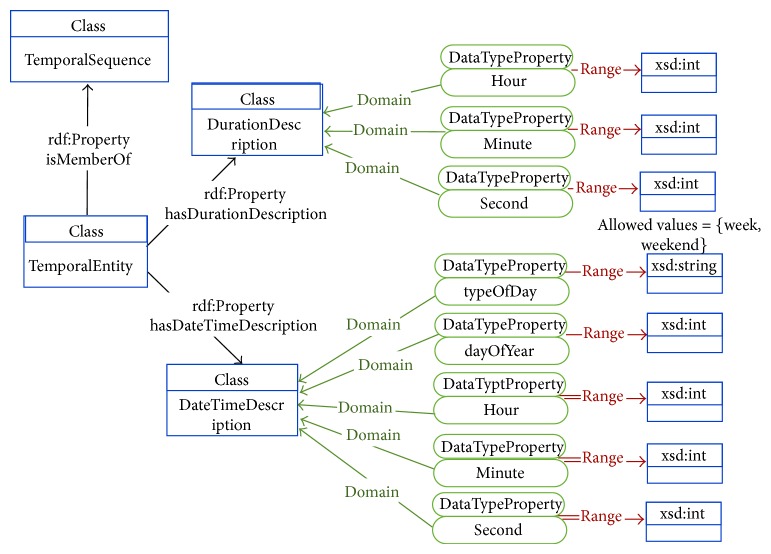
Conceptual diagram of the temporal information associated with the physical activity.

**Figure 5:**
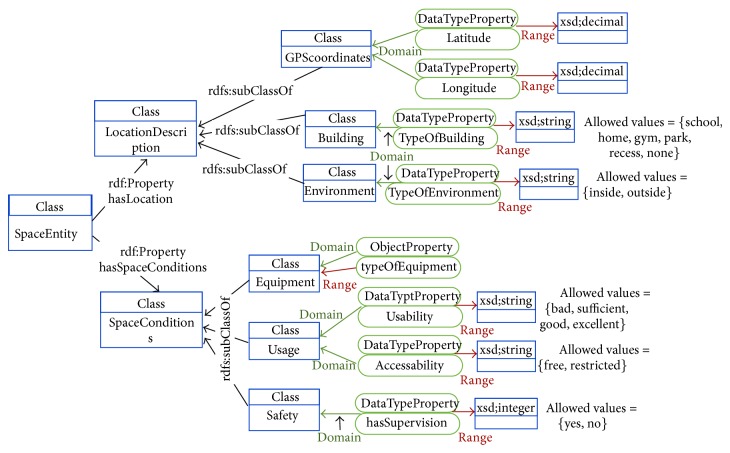
Conceptual diagram of information to characterize the space where the physical activity takes place.

**Figure 6:**
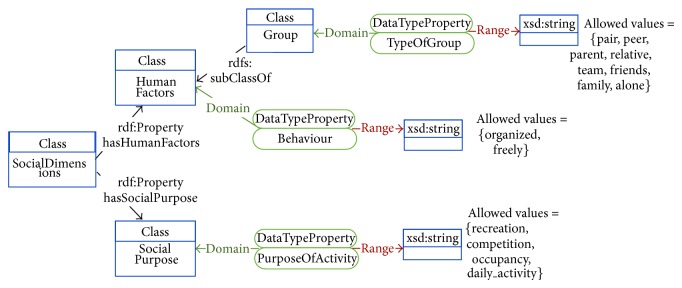
Conceptual diagram of information to characterize the social context associated with the physical activity.

**Figure 7:**
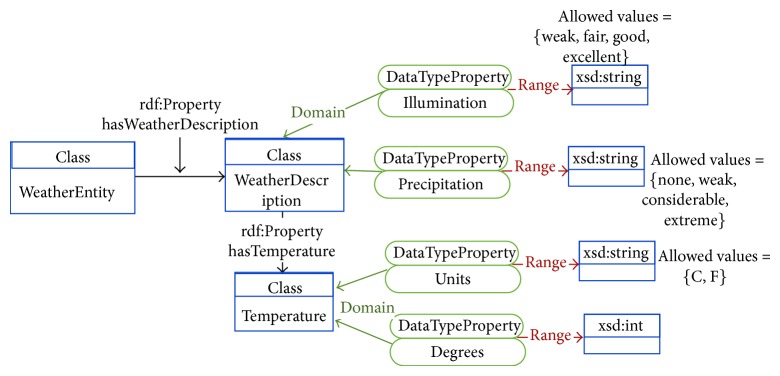
Conceptual diagram of information to characterize the weather context associated with the physical activity.

Each of the ontologies present in the OPA ontological model is further detailed in Figures 3–7.  Figure 3 represents the attributes of the individual performing the activity (the complete profile). Figures 4–7 provide illustrations of the classes in the extended ontologies that represent the time, space, social, and weather dimensions, respectively. 

We propose five main classes to characterize PA and SB because PA behaviors are affected by factors operating at several levels, which are broadly perceived as personal (such as biological and psychological attributes), social (family, affiliation group, and work factors, Figure 6), and environmental (contexts for different forms of activity and factors that could determine availability of relevant settings and opportunities, Figures 4, 5, and 7) ones [27]. Hence, these five classes provide a good compromise between simplicity and complexity (i.e., sufficient ability to make distinctions). While more complex models are possible, they would have little value if they could not be easily applied. The five main classes represent distinct contexts, and each has a clear utility for describing PA and SB. 

The models and concepts presented are in no way restrictive. Rather, we chose to define a set of base concepts and classes that can be flexibly and transparently extended, favored by the use of the proposed OPA ontology.

### 2.3. Applying the OPA Ontology

A principle adopted in the design of the OPA ontology is to reuse existing ontologies, whenever required; concepts already exist in other well-established ontologies. Instead of reinventing the wheel, this procedure has the additional benefit of facilitating the exchange of data between the knowledge base of the OPA system and other knowledge bases. The external ontologies that are integrated into the OPA ontology are well-known ontologies, referred to by the W3C. Together with the concepts specifically introduced by the OPA ontology, they provide all the concepts required to obtain a detailed characterization of people, their physical activity, and associated context.

(i) FOAF (Friend of a Friend) (http://xmlns.com/foaf/spec/): the FOAF ontology is used to express metadata about persons: their names, addresses, their interests, pictures of them, and so forth. FOAF is formally specified in RDFS/OWL and was developed within the context of the social Web, to enable establishing links between people on the Web. In our work, we use specifically the class “Person” and some of its properties and relationships with other (sub) classes. Including the FOAF ontology within the OPA framework allows us to describe the performers of (in)activity through a well-established specification. Nonetheless, given that FOAF was developed with the purpose of establishing social relationships among people on the Web, it does not provide all the properties needed to qualify the performer of an activity. As such, we have defined additional properties, which we use together with original FOAF slots to describe the instances of the class FOAF: Person.

(ii) REL (RELATIONSHIP: vocabulary for describing relationships between people) (http://vocab.org/relationship/.html, http://www.xml.com/pub/a/2004/02/04/foaf.html): the REL ontology provides a way to formally express relationships between persons. It is currently used in diverse applications in conjunction with the FOAF ontology. In our work we use it to express the relationships between participants in the physical activity.

(iii) OWL-Time (http://www.w3.org/TR/owl-time/) is the successor of DAML-Time ontology. It was developed by the W3C to formally express and relate temporal concepts, aiming at describing temporal aspects and properties of Webpages and Web services. 

To simulate the use of the OPA ontology, we will use the following example: “Catarina lives in Porto, Portugal, with her parents. Catarina's email address is catarina.costa@gmail.com. Catarina studies at the secondary school Garcia de Orta. She is on the 8th grade. She practices dance with some friends in a gym three times a week. Normally she walks everyday about 15 minutes in the morning to get to the school. In the afternoons when she has dance she also walks to the gym, which is pretty close to her home (5 minutes away). During the days when she does not have dance, Catarina enjoys walking for about 30 minutes with her mother, Maria Costa, in the evening, along the park near their home, whenever the weather is good.”

The OPA ontology can be used to describe this individual case and to enable systematic characterizations of the behavior. This requires creating instances of classes and providing values to the properties associated with the selected classes. Accordingly, the first step to formally describe the above case is to identify the required classes and the properties (or slots) associated with those classes that will be needed.

*(1)  Class FOAF: Person*

- an instance will be created to represent “Catarina Costa;”
- the slots “name”, “age,” and “race” will be filled in with appropriate values;
- the class.

*(2)  Class OPA: UserAttributes*

- given that the properties that exist in the ontology FOAF associated with the class Person are not sufficient for our purpose, an instance of the class UserAttributes, associated with the instance “Catarina Costa” through the relationship “has Attributes will be created;”
- one instance of each subclass of the UserAttributes class will be created (classes Anthropometry, Socio Economic Status (SES), and Psychosocial); corresponding slots will be filled in with appropriate values; it is assumed that these values are manually inserted or, otherwise, obtained automatically from a user profile database.

*(3)  Class OPA: Activity*

- given that four different activities are mentioned in the text, four instances of the class Activity will be created (“walkToSchool,” “Dance,” “walkToGym,” and “walkInPark”), related to the previous instance of Person, through the property “performsActivity”. Each instance of the class Activity will be further characterized by the 5 specific dimensions:
  (3.1) an instance of the class ActivityDescription to which it is connected through the property “hasActivityDescription”; each ActivityDescription instance has the subclasses “IntensityOfActivity”, “TypeOfActivity” and “BodyPart”; the latter is further characterized by the type of equipment manipulated during the activity;
  (3.2) an instance of the class TemporalEntity to which is connected through the property “hasTemporalEntity”, with associated subclasses instantiated and corresponding slots appropriately filled in. These sub-classes and slots will capture the temporal information present in the text, associated to each different activity performed by Catarina in two dimensions: the actual date when the activity is performed and the duration of the activity;
  (3.3) an instance of the class SpaceEntity to which is connected through the property “hasSpaceEntity”, with associated subclasses instantiated and corresponding slots appropriately filled in. These slots will capture the spatial information that is provided in the text, associated to each different activity performed by Catarina;
  (3.4) an instance of the class SocialEntity to which is connected through the property “hasSocialEntity”, with associated subclasses instantiated and corresponding slots appropriately filled in. These slots will capture the social aspects associated to each different activity performed by Catarina, which information is included in the text;
  (3.5) an instance of the class WeatherEntity to which is connected through the property “hasWeatherEntity”, with associated subclasses instantiated and corresponding slots appropriately filled in. These slots will capture the weather aspects associated to each different activity performed by Catarina, which information is included in the text.

*(4)  Class FOAF: Person*

- a second instance of this class will be created to represent Catarina's mother;
- the property “parentOf” borrowed from the REL ontology will be associated to this class.

*(5)  Class FOAF: Group*

- an instance of this class will be created to represent the dance group in which Catarina participates.

## 3. Conclusion

Future research directions in the PA and SB fields should lead to gain further insights into the levels and the contexts, under which they occur, that have stronger impact on those behaviors. The use of current and emerging technologies such as GPS and intelligent video processing is expected to be an integral part of this research field and provide richer information. The increased quantity and granularity that these new technologies can provide require additional tools to support its processing, management, and exchange.

The availability of the OPA ontology defining a common vocabulary for researchers in this area would provide an important tool for information sharing and reuse, thus increasing the opportunity for advances in the domain. The OPA ontology can enable automatically capturing the semantics of relationships that may exist between the different concepts and resources involved in PA and SB and their context in various dimensions (space, time, social, etc.). Accordingly they can greatly assist the researcher to infer new knowledge and conclusions derived from the captured knowledge and established relationships. Ontologies are being seen as the next level towards the fulfillment of the Semantic Web paradigm, to achieve interoperability and automated use of any resource available on the Web. Many disciplines now develop standardized ontologies that domain experts can use to share and annotate information in their fields [20].

The strength of this proposal is the support of the Compendium of Physical Activities [25]. The current paper presents a language and ontology consisting of a set of base classes but can be easily extended to accommodate additional information. By using the compendium, the OPA ontology can aggregate the qualitative information with the quantitative one allowing a better discrimination and characterization of the behavior observed, resulting in a more complete individual activity profile (iAP). 

It is our goal that this proposal will be well accepted by the researchers on this field and foster discussions and international development through the assistance of new technological tools. In view of the diversity between countries and populations in published work, results should be applicable to various settings and communities [27] since the ontology can incrementally become more flexible accommodating new information gathered from new scenarios. A systems' approach that focuses on populations and the complex interactions among the correlates of physical inactivity, rather than solely a behavioral science approach focusing on individuals, is the way forward to increase physical activity worldwide [10].
